# Risk Factors for Flares and New Lesions of Hidradenitis Suppurativa Following COVID-19 Disease: A Retrospective Cohort Study of 310 Patients in Greece

**DOI:** 10.3390/microorganisms13030542

**Published:** 2025-02-27

**Authors:** Aikaterini I. Liakou, Andreas G. Tsantes, Evangelia-Konstantina Bompou, Magdalini Kalamata, Efthymia Agiasofitou, Soultana Vladeni, Angeliki Dragoutsou, Konstantina A. Tsante, Petros Ioannou, Eleni Chatzidimitriou, Ourania Kotsafti, George Samonis, Georgia Vrioni, Stefanos Bonovas, Alexander I. Stratigos

**Affiliations:** 1 1st Department of Dermatology-Venereology, “Andreas Sygros” Hospital, Medical School, National and Kapodistrian University of Athens, 16121 Athens, Greece; 2 Laboratory of Haematology and Blood Bank Unit, “Attikon” Hospital, Medical School, National and Kapodistrian University of Athens, 12462 Athens, Greece; 3 Microbiology Department, “Saint Savvas” Oncology Hospital, 11522 Athens, Greece; 4 School of Medicine, University of Crete, 71003 Heraklion, Greece; 5 First Department of Medical Oncology, Metropolitan Hospital of Neon Faliron, 18547 Athens, Greece; 6 Department of Microbiology, Medical School, National and Kapodistrian University of Athens, 11527 Athens, Greece; 7 Department of Biomedical Sciences, Humanitas University, Pieve Emanuele, 20072 Milan, Italy; 8 IRCCS Humanitas Research Hospital, Rozzano, 20089 Milan, Italy

**Keywords:** hidradenitis suppurativa, epidemiology, infection, autoinflammatory disorders, immunology, cytokines, virology

## Abstract

Background: COVID-19 disease has been associated with flares or new onsets of various autoinflammatory diseases such as psoriasis and atopic dermatitis. Our aim is to investigate the occurrence and risk factors of flares or new onsets of Hidradenitis Suppurativa (HS) following COVID-19 disease. Methods: A retrospective cohort study was performed including 310 patients with HS following COVID-19 disease. Data on the rate of HS flares, new lesions, time of flare onset, and flare duration were recorded. Demographics, clinical characteristics, and treatment parameters were compared between patients with and without HS flares. Results: HS flares developed in 69 (22.2%) patients, with 14 experiencing their first episode. The median period between COVID-19 and flare onset was 17 days, with a median flare duration of 14 days. For new HS onset, the median period was 9.5 days, and the median duration was 13 days. Biologic treatment was less common in patients with flares (7.2% vs. 23.2%, *p* = 0.003), and fewer patients with flares were vaccinated (81.1% vs. 99.1%, *p* < 0.001). Multivariable analysis showed lower risk for flares in those receiving biologics (aOR = 0.14, *p* = 0.002) and those who were vaccinated (aOR = 0.02, *p* < 0.001). Conclusions: COVID-19 may trigger HS flares and new onset, with biologic treatment and vaccination offering protection.

## 1. Introduction

Severe acute respiratory syndrome coronavirus 2 (SARS-CoV-2) was spread rapidly throughout the world in 2019–2020, resulting in the Coronavirus Disease (COVID-19) pandemic [1]. Despite being mostly a respiratory disease, SARS-CoV-2 infection has been involved in various pathogenetic mechanisms, including a cytokine storm that has been associated with a hyperinflammatory environment in multiple organs. New onset of several autoimmune and autoinflammatory conditions has been reported following SARS-CoV-2 infection, while also exacerbation of preexisting autoinflammatory diseases was also evident in various studies [2,3,4,5].

Hidradenitis Suppurativa (HS) is a chronic inflammatory skin condition primarily affecting the apocrine gland-bearing areas of the body, such as the axillae, inguinal, and anogenital regions [6,7,8,9]. Given the immunological dysregulation seen in HS, concerns have been raised regarding the impact of COVID-19 infection in patients with HS [10,11]. Concerns about potential exacerbation of HS symptoms, adverse events, and the impact on disease activity and treatment response have been reported in the literature [12,13,14]. Existing evidence suggests that COVID-19 disease may affect the course of HS and lead to temporary disease exacerbation [15]. Studies investigating trigger factors for HS deterioration during the SARS-CoV-2 pandemic showed that 20% of HS patients experienced a worsening of their condition compared to before the pandemic. Interestingly, reported dietary changes during the pandemic were also associated, among other trigger factors, with HS exacerbation [16]. Moreover, persistent immune dysregulation and endothelial dysfunction after SARS-CoV-2 infection contribute to a systemic inflammatory state. This is further supported by the increased incidence of hypercoagulability and cardiovascular events in long-COVID patients [17]. Additionally, treatments widely used for severe COVID-19 infection, such as corticosteroids and remdesivir, might have influenced the course of autoinflammatory diseases [18,19]. It is true that the underlying mechanisms through which COVID-19 infection may trigger or worsen HS remain unclear, while it is crucial to differentiate between infection-related reactions and natural disease progression [20].

Moreover, there are reports that COVID-19 vaccination may also trigger HS flares or new HS-related lesions [21]. It is well established that COVID-19 vaccination activates the immune system. This is also evident from post-COVID-19 lymphadenopathy in both typical and atypical sites, as shown in many studies, reflecting an inflammatory response to vaccination [22,23]. On the other hand, the immune dysregulation associated with HS, including altered cytokine profiles and impaired immune cell function, raises concerns about the potential impact of HS on vaccine response. However, preliminary studies suggest that patients with HS can mount an adequate immune response to COVID-19 vaccination, with comparable antibody levels to the general population.

The aim of this study was to investigate the occurrence and risk factors of flares or new onsets of HS following COVID-19 disease, using real-world data.

## 2. Materials and Methods

A retrospective cohort study was performed from March 2020 to March 2024 at the HS outpatient clinic of our hospital. Written consent was obtained from all patients, while the study was approved by the Institutional Review Board of the “Andreas Sygros” Hospital for Venereal and Cutaneous Diseases (Reference No. 321/8-7-2024). Eligible patients were considered those who were ≥18 years old with a history of HS for more than one year and experienced COVID-19 disease (typical symptoms, confirmed by a positive rapid test and/or PCR). Those who developed a first episode of HS following COVID-19 disease were also included. Exclusion criteria were COVID-19 vaccination within 30 days from COVID-19 infection, frequent HS flares (≥1 flare/month) during the previous 12 months, severe or critical COVID-19 disease and chronic use of corticosteroids for other reasons, such as inflammatory bowel disease or rheumatoid arthritis. HS flare was defined as an episode of a new or substantial worsening of clinical signs or symptoms occurring within 30 days after the first symptoms of COVID-19 disease. The first day of “COVID-19 disease” was defined as the day when the first symptoms of the infection developed, even though confirmation (through rapid test and/or PCR) took place on a subsequent day. New HS-related lesions within 30 days following COVID-19 disease were diagnosed by specialized physicians based on widely used criteria. All patients experiencing HS flares had a positive response to the question: “Have you ever experienced a flare related to COVID-19-disease?”. Severe COVID disease was defined, according to widely used criteria, as having an SpO2 <93% on room air at sea level, a respiratory rate >30 breaths/min, a ratio of arterial partial pressure of oxygen to fraction of inspired oxygen (PaO2/FiO2) <300 mm Hg, or lung infiltrates >50%. Critical COVID-19 disease was defined as having respiratory failure, multiple organ dysfunction, or septic shock [24].

Evaluation of patients was performed using the Hurley score, the International Hidradenitis Suppurativa 4 (IHS4) score, and the Dermatology Quality of Life Index (DLQI). Moreover, patients were asked to self-evaluate the severity of their symptoms, on a scale from 1 to 10, with 10 being the worst HS flare they had ever experienced (Supplement S1). Data regarding demographics (such as age, gender, Body Mass Index [BMI]), HS scores (Hurley score, IHS4, DLQI), and type of treatment were collected and compared between patients with and without HS flares. Additional data such as time of flare/onset following COVID-19 disease and total duration of flare were also collected. Finally, patients’ self-reported score, Hurley score, IHS4, and DLQI were compared before and after COVID-19 disease.

### Statistical Analysis

Data were presented as means ± standard deviations (SD), medians with interquartile ranges (IQR) for quantitative parameters, or as frequencies (percentages) for qualitative parameters. The chi-square test and the two-sample Wilcoxon rank-sum (Mann–Whitney) test were employed to compare demographic and clinical characteristics between independent groups. To compare clinical parameters in patients with flares before and after COVID-19 disease, the Wilcoxon matched-pairs signed-rank test was used. Additionally, to evaluate the impact of biologic treatment and prior COVID-19 vaccination on the risk of developing HS flares, a multivariable logistic regression analysis was conducted. In this model, flare development served as the dependent variable, while gender, age, BMI, smoking status, biologic treatment, and prior COVID-19 vaccination were included as independent variables. Two-tailed *p*-values less than 0.05 were considered statistically significant. All analyses were performed using Stata 15.0 software (Stata Corp., College Station, TX, USA).

## 3. Results

**Figure 1:**
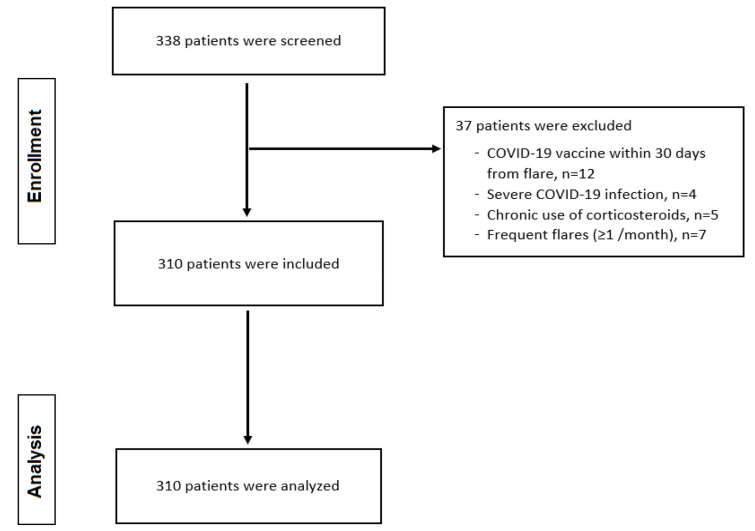
Flowchart of the study population.

Overall, 338 HS patients were assessed for their eligibility to be included in the study. Among them, 12 with HS flare following COVID-19 disease were excluded because they had received a COVID-19 vaccine within 30 days prior to the onset of the flare; therefore, the flare could be associated to the vaccination and not to COVID-19 disease [24]. Moreover, four patients were excluded because they suffered critical or severe COVID-19 illness, seven patients with frequent flares (≥1 flare/month), and five patients who were receiving corticosteroids on a chronic basis for other autoimmune diseases such as rheumatoid arthritis. Finally, 310 HS patients who had COVID-19 disease were included (Figure 1). In all these patients, COVID-19 disease was confirmed by a positive rapid test and/or PCR.

In the study population (n = 310), 69 patients (22.2%) had experienced HS flares after COVID-19 disease, while the majority (n = 241; 77.8%) did not have any flare. Among those patients who experienced HS flares (n = 69), there were 14 patients in whom HS was newly diagnosed. Therefore, 296 patients of our study population had been diagnosed with HS before COVID-19 disease. Patients with and without flares had comparable distributions of age (medians: 43 vs. 45 years; *p* = 0.20), BMI (medians: 28.6 vs. 30.3 kg/m^2^; *p* = 0.23), gender (males: 47.8% vs. 53.5%; *p* = 0.40), and smoking status (56.5% vs. 59.7%; *p* = 0.63) (Table 1). However, biologic treatment (anti-TNF therapy) was less common among patients with flares compared to those without (7.2% vs. 23.2%; *p* = 0.003), while also a lower percentage of patients with flares had been previously vaccinated for COVID-19 (81.1% vs. 99.1%; *p* < 0.001). Among the 61 patients who were treated with ant-TNF, 58 (95%) patients were vaccinated. Finally, patients with and without flares had comparable baseline disease scores, including the Hurley score, IHS4 score, and DLQI (*p* = 0.84, *p* = 0.79, and *p* = 0.28, respectively; Table 1).

The median period between COVID-19 disease and flare onset was 17 days (Interquartile Range [IQR]: 12–21), while these flares lasted for a median period of 14 days (IQR: 13–18; Table 2). Moreover, for those patients with HS flares following COVID-19 disease, the severity of symptoms based on the subjective self-reported score increased after COVID-19 disease (medians: 7 vs. 11; *p* < 0.001), while also the DLQI and the IHS4 scores indicated worsening of symptoms following COVID-19 disease (medians: 5 vs. 14; *p* < 0.001, and 6 vs. 16; *p* < 0.001, respectively; Table 2). Finally, the stage of HS based on Hurley score was also worse following COVID-19 disease, since most patients (n = 29, 42%) were on stage II before COVID-19 disease, while after COVID-19 disease, most patients (n = 38, 55%; *p* = 0.002) were on stage III.

The median time period between the COVID-19 disease and the onset of the first HS episode for the 14 patients with new HS onset was 9.5 days (IQR: 7–13 days), while the median duration of these first episodes was 13 days (IQR 11–18; Table 3).

The inverse association between biologic treatment (anti-TNF) and the risk of developing HS flares was further confirmed though the multivariable logistic regression analysis (adjusted odds ratio [aOR] = 0.14, 95% confidence interval [CI] 0.04–0.50; *p* = 0.002), indicating that biologic treatment may provide a protection against HS flares (Table 4). Similarly, prior COVID-19 vaccination was associated with a lower risk of HS flares following COVID-19 disease (aOR = 0.02, 95% CI 0.005–0.15; *p* < 0.001) (Table 4).

## 4. Discussion

There is some evidence indicating that COVID-19 infection is associated with flares of autoinflammatory diseases [12]. COVID-19 infection may cause a cytokine storm that can exacerbate diseases with inflammation-based pathophysiology. The aim of this study was to investigate the association between COVID-19 infection and exacerbations of HS. Our results indicate that COVID-19 infection may cause an HS exacerbation in almost 20% of patients. Moreover, new HS lesions were evident in some patients, suggesting that COVID-19 infection may also result in a new HS onset. The median periods between COVID-19 infection and HS flares, and between COVID-19 infection and HS new onset were 17 and 9.5 days, respectively, whereas the median durations of HS flares and first HS episodes were 14 and 13 days, respectively. Moreover, patients who were on biologic treatment (anti-TNF) for HS and those who were previously vaccinated for COVID-19 infection were less likely to develop HS flares following disease. These findings suggest that COVID-19 vaccination and biologic treatment (anti-TNF) may have a protective role against HS flares. To our knowledge, this is the first cohort study investigating the impact of COVID-19 infection on the natural course of HS.

Our findings are in line with other studies indicating that COVID-19 infection is associated with various autoimmune diseases such as rheumatoid arthritis, immune mediated thrombocytopenia, multiple sclerosis, and vasculitis [25]. In a recent retrospective cohort study including 362 patients with psoriatic arthritis, rheumatoid arthritis, and ankylosing spondylitis, 117 (32.3%) patients were affected by COVID-19 infection with 40 (34.2%) of them experiencing an inflammatory arthritis flare within one month of the infection, while 3 (7.5%) of them needed to switch to another type of therapy [26]. In our study, HS flares following COVID-19 infection were also developed within one month (median time: 17 days) after infection. In another recent study evaluating 1928 patients with rheumatoid arthritis following COVID-19 infection, it was shown that younger age, past history of tuberculosis, treatment with methotrexate, and poor global physical health were independent factors associated with disease flares in patients [27]. As opposed to the results of this study, gender, smoking, and BMI were similar between patients with or without HS flares in our study. Finally, the authors of the review article enrolling 3,335,084 individuals reported an increased incidence of inflammatory arthritis, including rheumatoid arthritis, after COVID-19 infection, with the greatest increase occurring during the first year following COVID-19 infection [28].

There are also several studies evaluating the association between flares and COVID-19 infection in patients with autoimmune skin disorders such as HS. In a recent meta-analysis including 185,000 psoriasis patients, it was found that patients who had not missed their scheduled biologic administration were experiencing milder COVID-19 symptoms. The authors of this meta-analysis reported that biologics may have a desirable effect on overcoming the hyperinflammation state caused by COVID-19 infection and recommended against withholding biologics due to COVID-19 infection [29]. Several studies enrolling large populations have also been conducted in order to assess the impact of COVID-19 infection on psoriasis course. One questionnaire-based study in the Netherlands assessed 1132 adult patients with atopic dermatitis and psoriasis and found that 26% of them experienced worsening of their condition during symptomatic infection with SARS-CoV-2 [30]. Indeed, atopic dermatitis is another inflammatory skin disease that has been reported to be affected after COVID-19 infection. In a study evaluating 21 adults with atopic dermatitis who had been infected with SARS-CoV-2, it was found that 43% of the patients experienced disease exacerbation, although it did not require systemic intervention. Moreover, patients with severe atopic dermatitis who were receiving immunosuppressive therapy had milder exacerbations [31].

The exact pathophysiology involved in the association between COVID-19 infection and flares of autoinflammatory diseases is still unknown. It has been reported that other viruses such as human papillomavirus, hepatitis B, and influenza can also contribute to the onset or worsening of autoimmune disorders through molecular mimicry. This refers to the resemblance between certain viral components and self-molecules, leading to the activation of the immune system against pathogenic antigens and the mistaken attack on similar proteins [32]. Other evidence suggests that viral-induced autoimmunity can be triggered by various mechanisms including bystander activation and immortalization of infected B cells [33]. The term “cytokine release syndrome” describes the uncontrolled secretion of pro-inflammatory cytokines induced by COVID-19 infection, mostly interleukins and tumor necrosis factor α, which disrupt human innate and acquired immune response [34,35]. Multiple autoantibodies have also been detected in patients with COVID-19 infection, including anti-CCP antibodies (biomarkers for psoriasis/inflammatory arthritis) and anti-nuclear antibodies (biomarkers for Guillain–Barré syndrome) [34].

The management of biologic treatment in patients with COVID-19 infection has been a topic of debate, with concerns regarding the potential negative impact of biologic agents on the course of COVID-19 infection. COVID-19 infection is a multiphasic viral disease which commences with an antiviral response phase followed by a hyperinflammatory state. Many cytokines such as tumor necrosis factor-alpha (TNFa), interleukin (IL)-1, and IL-6 are related to the cytokine storm that influences the severity of COVID-19 infection [36]. The main cytokines that dominate the first (antiviral) phase differ from the cytokines which prevail during the hyperinflammatory phase [14]. Interleukin (IL)-15, interferon-α, interferon-β, and interferon-γ are the major cytokines responsible for viral clearance, whereas TNF-α, IL-17, IL-6, and granulocyte–monocyte colony stimulating factor preponderate during the hyperinflammatory phase [37,38]. Therefore, it seems reasonable not to discontinue anti-TNF-α and anti-IL-17 medications during the hyperinflammatory state of COVID-19, since these medications do not seem to affect the course of the antiviral phase. In phase 3 trials of adalimumab for HS, it was shown that there is a slightly escalated risk for total infections and nasopharyngitis by 2.5% but there was no significant difference between adalimumab and the placebo group in terms of upper respiratory tract infections [39]. The authors of a recent study showed that treatment of HS with the adalimumab does not increase the risk for COVID-19 infection [10]. This is in line with our findings, since this is the first study –to our knowledge- showing that post COVID-19 HS flares were more likely to occur in patients who were not receiving biologic agents, indicating a protective role of these medications against HS flares.

Following the launch of COVID-19 vaccination, cases of newly diagnosed autoimmune disorders had been reported, including autoimmune liver diseases, GBS, IgA nephropathy, and Hidradenitis Suppurativa, as potential side effects of COVID-19 vaccination [21,24]. It has been hypothesized that COVID-19 vaccination can activate autoimmune diseases through the production of autoantibodies (e.g., platelet factor 4 antibody-mediated platelet may lead to immune mediated thrombocytopenia). Moreover, vaccine adjuvants can enhance the immune response through activation of the NLR pyrin domain containing 3 (NLRP3) inflammasome, which is part of the innate and adaptive immune system and is linked to a range of autoimmunity. This is in line with a previous study from our group showing that COVID-19 vaccination may be associated with HS flares, since HS flares occurred in 19.2% of vaccinated patients. However, these post-vaccination flares were less severe (based on DLQI and ISH4 scores) compared to those that were observed in this study following COVID-19 infection. Moreover, another interesting finding of our study is that patients who were previously vaccinated against COVID-19 infection were less likely to develop flares. Therefore, based on these findings, COVID-19 vaccination should be strongly recommended in HS patients, not only because post-vaccination flares are less severe compared to post COVID-19 flares, but also because in the long-term, vaccination may have a protective role against future flares following COVID-19 infection.

Our study has some limitations that need to be mentioned. First, it was a single center study with no control group; therefore, comparison of flare rates between HS patients with and without COVID-19 infection could not be performed. Therefore, comparative studies with larger populations are required to validate our findings. Moreover, the relatively small number of patients poses a certain limitation, although this is the first study evaluating patients with HS flares following COVID-19 disease. Also, the definition of HS flare that was used in our study may lead to over- or under-estimation of the true incidence of HS flares due to COVID-19 infection. HS flares were defined as those that occurred within one month from the infection. However, a flare within one month could be attributed to the natural history of the disease, while on the other hand, a flare caused by COVID-19 infection can also occur later than a month. Moreover, the questionnaire that was used to evaluate the severity of HS symptoms, although standard, has not been validated which limits its reliability. Another limitation is that the severity of the COVID-19 disease was not evaluated; therefore, an association between the severity of the infection and the risk of developing HS flares could not be investigated. Last, available data regarding other comorbidities or dermatological conditions that could also be associated with the risk of developing HS flares were not available for our study population.

The COVID-19 pandemic has raised challenges in the management of chronic autoinflammatory diseases, such as HS. Physicians should be prepared to treat disease exacerbations following COVID-19 disease, since as indicated from our findings, about one out of five patients with HS may experience a flare following COVID-19 disease. However, HS flares were more likely to develop in patients who were not vaccinated, indicating that COVID-19 vaccination should be strongly encouraged in HS patients in order to reach an increased rate of immunity against SARS-CoV-2 virus. The biologic medications should also not be discontinued during COVID-19 infection, since based on our findings, they seem to have a protective role in post-COVID-19 HS flares. Although the worst has passed with the COVID-19 pandemic, our results regarding the association between COVID-19 disease and HS flares could be the basis for further research regarding flares of autoinflammatory diseases following viral infections, while they could aid physicians to be better prepared in case of upcoming challenges for global health with new infectious pathogens.

## Figures and Tables

**Table 1:** Characteristics of the study population (n = 310).

Parameters	Patients with Flares (n = 69)	Patients Without Flares (n = 241)	*p*–Value
Gender (male, %)	33 (47.8)	129 (53.5)	0.40
Age (years)	41.0 ± 11.6, 43.0 (31.0–49.0)	43.5 ± 12.6, 45.0 (37.0–52.0)	0.20
BMI (kg/m^2^)	30.2 ± 8.0, 28.6 (23.5–35.0)	30.6 ± 6.6, 30.3 (26.1–33.6)	0.23
Smoking Status	39 (56.5)	144 (59.7)	0.63
Vaccination	56 (81.1)	239 (99.1)	**<0.001**
Vaccine Type mRNA non–mRNA	51 (73.9) 5 (7.2)	200 (82.9) 39 (16.1)	0.16
Baseline Hurley score I II III	15 (21.7) 29 (42.0) 25 (36.2)	54 (22.4) 92 (34.2) 95 (35.3)	0.84
Baseline IHS4 score	8.0 ± 7.2, 6 (3–11)	7.8 ± 6.5, 6 (3–12)	0.79
Baseline DLQI score	6.4 ± 5.0, 5 (3–9)	7.5 ± 5.8, 6 (3–11)	0.28
Biologic treatment (anti-TNF)	5 (7.2)	56 (23.2)	**0.003**

The data are presented as means ± standard deviations (SD), medians with interquartile ranges (IQR) for quantitative parameters, or as frequencies (percentages) for qualitative parameters. The Wilcoxon rank-sum and the chi-square tests were used for comparisons between the two groups. Abbreviations: BMI, Body Mass Index; DLQI, Dermatology Life Quality Index; IHS4, International Hidradenitis Suppurativa Severity Score System.

**Table 2:** Characteristics of HS patients with flares or new-onset disease following COVID-19 (n = 69).

Parameters	Before COVID-19	After COVID-19	*p*-Value
Time of onset of flare (days)	16.2 ± 5.9, 17 (12–21)		-
Duration of flares (days)	15.4 ± 4.3, 14 (13–18)		-
New locations	51 (20.4)		-
Patients’ reported score	6.7 ± 1.7, 7 (6–8)	10.7 ± 1.7, 11 (10–12)	**<0.001**
Hurley score I II III	15 (21.4) 29 (42.0) 25 (36.2)	2 (2.9) 29 (42.0) 38 (55.0)	**0.002**
IHS4 score	8.0 ± 7.2, 6.0 (3–11)	17.4 ± 8.0, 16 (11–22)	**<0.001**
DLQI score	6.4 ± 5.0, 5 (3–9)	15.8 ± 7.2, 14 (10–26)	**<0.001**

The data are presented as means ± standard deviations (SD), medians with interquartile ranges (IQR) for quantitative parameters, or as frequencies (percentages) for qualitative parameters. The Wilcoxon signed-rank test was used for the comparison of scores before and after vaccination. Abbreviations: DLQI, Dermatology Life Quality Index; IHS4, International Hidradenitis Suppurativa Severity Score System.

**Table 3:** Characteristics of the 14 patients with the new onset of HS disease.

Parameters	
Gender (male, %)	7 (50.0)
Age (years)	42.5 ± 14.2, 42 (30–53)
BMI (kg/m^2^)	30.5 ± 7.0, 29.7 (25.1–34.1)
Smoking Status	7 (50.0)
Time of onset after disease (days)	10.0 ± 3.8, 9.5 (7–13)
Duration of first episode (days)	14.2 ± 4.9, 13 (11–18)
Vaccinated for COVID	12 (85.7)
Biologic treatment (anti-TNF)	5 (35.7)
Patients’ reported score	10.2 ± 1.6, 10 (9–11.5)
Hurley score I II III	0 (0.0) 8 (57.1) 6 (42.9)
IHS4 score	14.0 ± 5.0, 13.5 (10–18)
DLQI score	10.3 ± 6.4, 9 (7–11)

The data are presented as means ± standard deviations (SD), medians with interquartile ranges (IQR) for quantitative parameters, or as frequencies (percentages) for qualitative parameters. Abbreviations: DLQI, Dermatology Life Quality Index; IHS4, International Hidradenitis Suppurativa Severity Score System.

**Table 4:** Multivariable logistic regression analysis: flare development served as the dependent variable, while gender, age, BMI, smoking status, biologic treatment, and prior COVID-19 vaccination were included as independent variables.

Variables	Development of Flare		
	aOR	(95% CI)	*p*-Value
Gender (male)	0.69	(0.28–1.68)	0.42
Age (per 1-year increase)	0.99	(0.95–1.004)	0.11
BMI (per 1-unit increase)	1.01	(0.95–1.08)	0.57
Vaccination	0.02	(0.005–0.15)	**<0.001**
Biologic treatment (anti-TNF)	0.14	(0.04–0.50)	**0.002**
Smoking status	0.69	(0.34–1.39)	0.30

Abbreviations: aOR, adjusted odds ratio; CI, confidence interval.

## Data Availability

The raw data supporting the conclusions of this article will be made available by the authors on request.

## Supplementary Materials

The following supporting information can be downloaded at https://www.mdpi.com/article/10.3390/microorganisms13030542/s1. Supplement S1. Questionnaire distributed to patients, asking about possible exacerbation or new lesions of Hidradenitis Suppurativa, following COVID-19 disease.

## Abbreviations

The following abbreviations are used in this manuscript:

COVID-19	Coronavirus Disease 2019
HS	Hidradenitis Suppurativa
BMI	Body Mass Index;
DLQI	Dermatology Life Quality Index;
IHS4	International Hidradenitis Suppurativa Severity Score System.
aOR	Adjusted odds ratio
CI	Confidence interval
